# Greenhouse Gas Emission Fluxes in Urban Wetlands of Qinghai–Tibet Plateau

**DOI:** 10.3390/biology15110871

**Published:** 2026-05-31

**Authors:** Jianhua Si, Jiawen Kang, Shipeng Zhou, Jiawei Tian, Qilian Xie, Zhiwei Chen, Yue Qi, Qi An, Yanhong Gong, Biyu Qin, Sujin Lu

**Affiliations:** 1 College of Forestry and Grassland, Qinghai University, Xining 810016, China; sijianhua1@163.com (J.S.); xieqilian123456@126.com (Q.X.); 18846792640@163.com (Z.C.); 2 College of Eco-Environmental Engineering, Qinghai University, Xining 810016, China; kjw160125@163.com (J.K.); zspengz@163.com (S.Z.); 18509713891@163.com (J.T.); shengtaiqiyue@qhu.edu.cn (Y.Q.); 18009710606@163.com (Q.A.); gongyh15@163.com (Y.G.); qby13608631905@163.com (B.Q.)

**Keywords:** plateau urban wetland, greenhouse gas, RDA analysis, CH_4_, CO_2_, N_2_O

## Abstract

Background: This study aims to measure Greenhouse Gas (GHG) emission fluxes at the soil–air and water–air interfaces in urban wetlands on the Qinghai–Tibet Plateau and identify the primary controlling factors. The objective is to elucidate the key drivers of carbon and nitrogen processes at different interface levels in wetlands within high-altitude urban settings, thereby providing a scientific basis for accurately estimating their contribution to greenhouse gas emissions. Results: In the wetlands of Xining City, with the exception of soil pH, bulk density, and moisture content (which showed no significant change over time), all other soil physicochemical properties differed significantly among the three wetlands and among the sampling periods (*p* < 0.05). Soil moisture content was less affected by variations across different wetlands and over time, and differences in soil physicochemical properties among different wetlands were small (*p* > 0.05). Significant differences were observed in the spatiotemporal variations in the physicochemical properties of water bodies in Xining’s wetlands (*p* < 0.05), although water pH and total organic carbon (TOC) were less affected by the interaction between different wetlands and time periods. There were no significant differences in the bulk density and moisture content of wetland sediments in Xining over time (*p* > 0.05), while all other physicochemical indicators of sediments showed significant differences (*p* < 0.05). The physicochemical properties of sediments were influenced by both different wetland types and different time periods. GHG fluxes at the water–air interface in Xining wetlands were greater than those at the soil–air interface; overall, GHG emissions from both interfaces acted as “sources.” Seasonal variations in wetland GHG emissions were pronounced, with emission peaks occurring from June to August. The study found that the primary soil factor influencing GHG emissions at the soil–air interface was total phosphorus (TP), while the primary sediment factors affecting emissions at the water–air interface were TP and nitrate nitrogen (NO_3_^−^-N), and the primary water factor was TOC. The interannual cumulative emissions from both interfaces in the wetland totaled 705.88 g·m^−2^. GHG emissions from the soil–air and water–air interfaces contributed 47.88% and 52.12%, respectively, to the global warming potential (GWP) of the wetland, while methane (CH_4_), carbon dioxide (CO_2_), and nitrous oxide (N_2_O) contributed 32.55%, 62.33%, and 5.12%, respectively, to the GWP. Conclusions: Investigating the GHG emission patterns in Xining’s wetlands and identifying the primary factors influencing these emissions provides a scientific basis for the protection and restoration of these wetlands. This is of great significance for safeguarding the ecological security of Xining’s wetlands as well as the overall ecological security of high-altitude wetlands.

## Simple Summary

Urban wetlands on the Qinghai–Tibet Plateau play an important role in regulating greenhouse gas emissions and maintaining ecological functions in high-altitude cities. However, little is known about how different types of urban wetlands influence greenhouse gas release under plateau environmental conditions. In this study, greenhouse gas emission fluxes from the water–air and soil–air interfaces were investigated in three typical urban wetlands in Xining City, including riverine, constructed, and semi-constructed wetlands. The results showed clear spatial and seasonal differences in carbon dioxide, methane, and nitrous oxide emissions among the wetlands. Environmental factors such as nutrient levels, temperature, and moisture conditions strongly affected greenhouse gas emissions. Constructed wetlands influenced by urban activities generally showed higher greenhouse gas emissions than relatively natural wetlands. This study improves understanding of greenhouse gas dynamics in plateau urban wetlands and provides scientific support for wetland management, ecological restoration, and low-carbon urban development in high-altitude regions.

## 1. Background

In recent years, research attention on N_2_O emissions, a potent greenhouse gas with a GWP 298 times that of CO_2_ (the Fourth Assessment Report of the Intergovernmental Panel on Climate Change, IPCC), has increased. Nitrification and denitrification processes in wetland soils are the main sources of N_2_O emissions [1]. Additionally, decomposition of soil organic matter is considered the primary source of CO_2_ and CH_4_ emissions. The anaerobic conditions in wetlands promote CH_4_ production, with soil temperature and moisture content serving as critical regulatory factors [2].

Xining City is located in the eastern Qinghai–Tibet Plateau. Under the influence of its high-altitude geographical location, cold and dry climate conditions, and regional human activities, the wetlands here exhibit unique characteristics of sensitivity and vulnerability. Greenhouse gas emission fluxes in these plateau urban wetlands differ significantly from those in lowland ecosystems due to distinct environmental conditions [3]. Notably, “plateau cities” refer to urban areas situated at high altitudes-typically above 2000 m-including cities on the Qinghai–Tibet Plateau (e.g., Xining, Lhasa) and the Yunnan-Guizhou Plateau (e.g., Kunming, Lijiang). Currently, relatively few studies have examined greenhouse gas emission fluxes from wetlands in plateau cities. Scholars worldwide recognize that key factors such as dissolved oxygen (DO), ammonia nitrogen (NH_3_-N) concentration, soil organic carbon (SOC), dissolved organic carbon (DOC), water and air temperature, pH, wind speed, and water depth jointly regulate greenhouse gas emissions from wetlands and act as primary drivers of their spatial and temporal variability [4,5,6,7,8]. An in-depth study on greenhouse gas emission flux changes in Xining City’s wetlands provides an effective approach for rational utilization and protection of wetland resources in plateau cities and offers a scientific basis for the protection and restoration of alpine wetlands.

Therefore, the primary objectives of this study are: (1) to quantify CH_4_, CO_2_, and N_2_O emission fluxes at the soil–air and water–air interfaces in urban wetlands, using Xining, a city on the Qinghai–Tibet Plateau, as a representative case study; (2) to identify the key physicochemical factors (soil, water, and sediment properties) that control these emissions; and (3) to estimate the cumulative GHG emissions and their GWP. The hypotheses to be tested include: (H1) GHG emission fluxes exhibit significant seasonal and spatial variations, with summer fluxes higher than those in other seasons, and emissions from semi-natural wetlands higher than those from riverine or artificial wetlands; (H2) The water–air interface is a stronger source of CH_4_ and N_2_O emissions, while CO_2_ exhibits the opposite pattern (higher at the soil–air interface); (H3) TP in soil, TOC in water, and NO_3_^−^-N in sediments are the primary drivers of GHG emissions across different interfaces.

## 2. Materials and Methods

### 2.1. Overview of the Study Area

**Figure 1:**
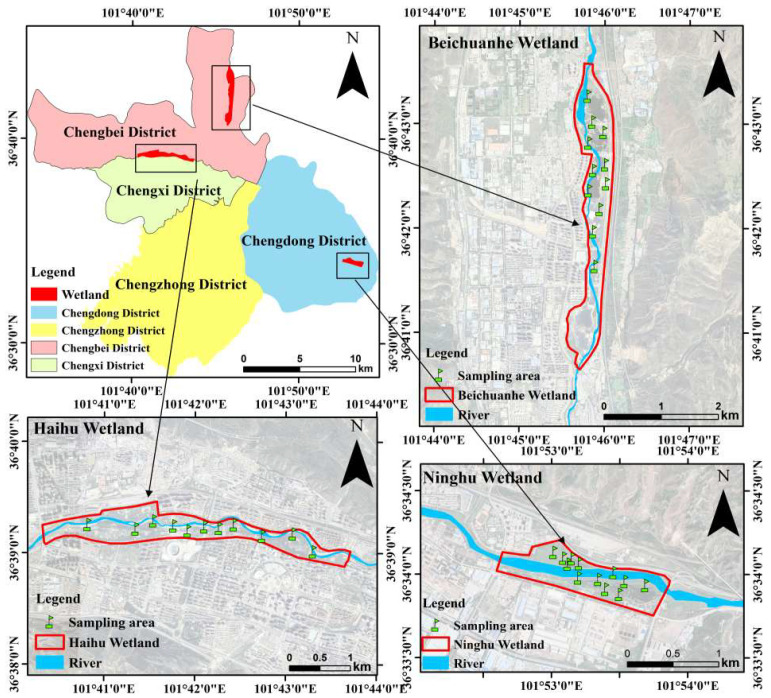
Geographical map of the study region.

The Huangshui National Wetland Park comprises five major wetland parks: Huoshaogou Wetland, Haihu Wetland, Beichuan Wetland, Ninghu Wetland, and the Huangshui River Main Channel Park. This study focuses on riverine wetlands (Beichuanhe Wetland), constructed wetlands (Ninghu Wetland), and semi-constructed wetlands (Haihu Wetland) [9]. The Beichuanhe Wetland is located in the core section of the Beichuanhe in Chengbei District, Xining City, with a total area of approximately 173.5 ha. The dominant aquatic plant species are common reed (*Phragmites australis* (Cav.) Trin. ex Steud.) and Oriental cattail (*Typha orientalis* C.Presl). The Ninghu Wetland is situated in Chengdong District, Xining City, with a total land area of approximately 85.5 ha. It extends from the newly constructed bridge on Guinan Road in the west to Xiaxiaokou in the east. The dominant aquatic plant species are common reed (*Phragmites australis* (Cav.) Trin. ex Steud.) and Oriental cattail (*Typha orientalis* C.Presl), along with water sedge (*Schoenoplectus tabernaemontani* (C.C.Gmel.) Palla) and water lily (*Nymphaea* sp.), among others. The Haihu Wetland is located in Chengxi District, Xining City, with a total area of approximately 146 ha. It extends from the Huangshui Bridge in the west to Huoshaogou in the east [10]. Aquatic plants include common reed (*Phragmites australis* (Cav.) Trin. ex Steud.), cattail (*Typha orientalis* C.Presl), and water lily (*Nymphaea* sp.), among others [11]. The geographical locations of the wetlands and sampling sites are shown in Figure 1. Plant nomenclature follows the Flora of China (http://www.iplant.cn/foc), accessed on 20 July 2024.

Although all three wetlands are located within Xining City, they differ substantially in hydrological conditions, nutrient inputs, surrounding land use, and levels of anthropogenic disturbance [12]. The Beichuanhe Wetland is relatively close to a natural riverine wetland with stronger hydrological connectivity and lower nutrient loading. In contrast, Ninghu Wetland is a constructed wetland influenced by urban runoff and artificial hydrological regulation, while Haihu Wetland represents a semi-constructed wetland with intermediate environmental characteristics [13]. These differences provide an ideal framework for comparing greenhouse gas emission dynamics among wetlands under different management and disturbance conditions. Detailed characteristics of the three wetlands are summarized in Table 1.

### 2.2. Research Methods

Experimental design summary: The study compared greenhouse gas emissions among three wetland types with different disturbance levels: Beichuanhe (near-natural reference), Ninghu (constructed), and Haihu (semi-constructed). Spatial replicates: three fixed sampling points per wetland (*n* = 3 per month). Temporal replicates: monthly measurements over three consecutive days (averaged) for 24 months. Technical replicates: GHG fluxes calculated from linear regression of four gas samples (0, 10, 20, 30 min; R^2^ > 0.95). Control: Beichuanhe served as a reference to assess anthropogenic disturbance effects.

#### 2.2.1. Research on Wetland Environmental Factors

(1) Collection of Wetland Soil Samples

Based on field surveys of wetlands in Xining, the depth of the riparian zone soil varied between approximately 1 and 2.5 m depending on water conditions; therefore, the soil collection area was designated within 3 m of the riverbank. From April to November in 2024 and 2025, three soil plots were randomly selected in the east, west, and south directions, respectively. Soil samples were collected from 10 m × 10 m plots, resulting in a total of 109 soil samples collected during the study period. Within the designated soil plots, a ring cutter was used to collect soil samples at a depth of 0–10 cm. Additionally, 3–5 representative sampling points were randomly selected within each plot. The collected soil samples were placed in self-sealing bags, transported to the laboratory, and allowed to air-dry naturally prior to analysis.

(2) Collection of Wetland Water Samples

From April to November 2024–2025, wetland water samples were collected in accordance with the Technical Specifications for Surface Water Environmental Quality Monitoring (HJ91.2-2022). Overlying water samples were collected randomly using 500 mL brown sterile glass bottles from vegetation-covered water areas near different sampling points in each wetland. Three bottles were collected at each sampling point, with a sampling depth of 10 cm below the water surface. A total of 102 water samples were collected during the study period. While collecting water samples, the depth of the sampling points was measured using a ruler. After adding a fixative, the samples were placed in an insulated box containing ice packs and transported back to the laboratory for analysis. The temperature at each sampling point was recorded using a thermometer.

(3) Collection of Wetland Sediment Samples

From April to November 2024–2025, following the wetland sediment sampling method, and in reference to the “Technical Specifications for Soil Environmental Monitoring” (HJ 166-2004), three surface sediment samples were collected from a depth of 0–10 cm at each sampling point using a columnar sediment sampler. The three samples were mixed, and 500 g of the mixture was placed into a self-sealing plastic bag (140 mm × 200 mm), sealed, and transported back to the laboratory for the determination of Total nitrogen (TN), TP, NH_3_-N, NO_3_^−^-N, bulk density, and other parameters. A total of 102 sediment samples were collected during the study.

#### 2.2.2. Collection of Greenhouse Gas Samples from Wetlands

From January 2024 to December 2025, three GHG sampling points were established at each wetland in Xining City. Sampling points were located away from areas of intensive human activity and were generally distributed across the upper, middle, and lower sections of the wetland systems. Sampling was conducted 1–2 times per month on clear and low-wind days. Measurements were performed from 9:00 a.m. to 8:00 p.m. over three consecutive days each month to capture daytime variability in GHG emissions. The averaged values were used to estimate representative daytime GHG fluxes during the sampling period, following commonly adopted static chamber protocols in wetland GHG studies [14].

**Figure 2:**
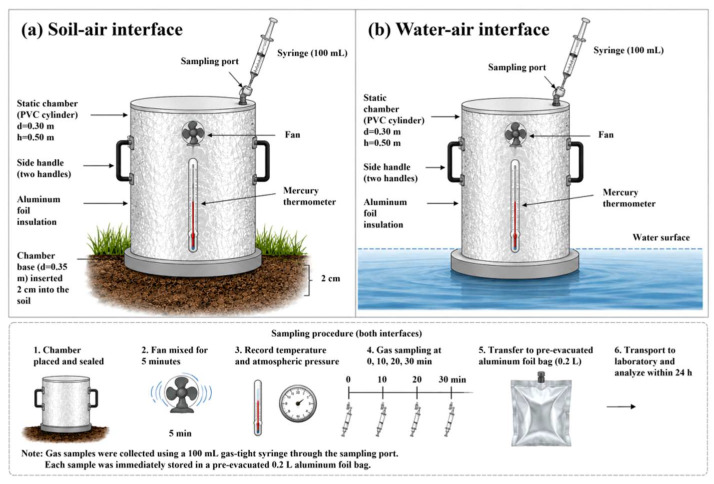
Illustration of greenhouse gas sampling. Panels: (**a**) Soil-air interface, (**b**) Water-air interface.

GHG samples at the soil–air interface were collected using a custom-made static chamber. The static chamber consisted of a base and a chamber body, equipped with a small fan and a thermometer. The chamber body was a PVC cylinder with a diameter of 0.3 m and a height of 0.5 m. The outer layer of the chamber was covered with an aluminum foil thermal insulation film to prevent rapid temperature changes during sampling from affecting the results. The base of the static chamber was pre-installed at each wetland site. The base (d = 0.35 m) was inserted 2 cm into the soil layer and leveled with the ground surface. During sampling, the chamber was connected to the base and sealed. Vegetation and debris were removed from the surface, and the fan inside the chamber was turned on for 5 min to mix the air. Simultaneously, readings from the thermometer and barometer were recorded; a barometer was used to record ambient atmospheric pressure for subsequent flux calculations (see Formula (1)). Thereafter, a 100 mL syringe was used to draw gas samples from the chamber at 0, 10, 20, and 30 min, injecting them into 0.2 L vacuum aluminum foil bags for transport to the laboratory, where analysis was conducted within 24 h. For GHG sampling at the water–air interface, the static dark chamber was floated on the water surface and secured in place, ensuring the base is level with the liquid surface. The sampling method was the same as for the soil–air interface, and thermometer and barometer readings were recorded simultaneously. Figure 2 shows a schematic diagram of GHG sampling.

### 2.3. Sample Analysis

Soil samples were air-dried, and after removing foreign matter such as stones and roots, they were ground and sieved through 60-mesh and 100-mesh screens, respectively, for storage. Soil bulk density and moisture content were determined using the ring-knife method. In situ soil samples were collected using a 100 cm^3^ ring-knife; after weighing the fresh weight on-site, the samples were dried at 105 °C to constant weight, and the dry weight was recorded. Bulk density (g·cm^−3^) was calculated as the mass of oven-dried soil (g) divided by the volume of the ring cutter (cm^3^); moisture content (%) was calculated as ((fresh soil weight − oven-dried soil weight)/(oven-dried soil weight)) × 100. The pH of soil samples was measured using a pH meter after extraction. A portion of the soil samples was crushed, ground, and sieved through a 100-mesh sieve; total carbon (TC) and total inorganic carbon (IC) were then determined using a total organic carbon analyzer (TOC-L; Shimadzu, Kyoto, Japan), and the TOC content was calculated as the difference between the two. The remaining samples were analyzed using a flow chemistry analyzer (AA500; SEAL Analytical, Norderstedt, Germany). Key physicochemical indicators of the soil included NH_3_-N, NO_3_^−^-N, TP, and TN. Collected wetland water samples were transported to the laboratory, filtered using an automatic filtration system with 0.45 µm pore size filters, and the resulting filtrate was stored in polyethylene bottles at −20 °C. Sample pretreatment was completed within 8 h of collection. The main physicochemical parameters of the water samples included NH_3_-N, NO_3_^−^-N, TP, and TN. A TOC-L analyzer (Shimadzu, Kyoto, Japan) was used to determine TOC content in the water samples. The bulk density and moisture content of sediments were primarily determined using the drying method. Pre-frozen sediment samples were dried using a freeze dryer (FDU-5500A; EYELA, Tokyo Rikakikai Co., Ltd., Tokyo, Japan). The determination of parameters for freeze-dried sediment samples followed the same procedures as for soil samples. The methods for determining parameters in soil, water, and sediment samples, as well as greenhouse gas measurements, were summarized in Table 2.

### 2.4. Data Calculation

(1) Greenhouse gas emission flux

GHG emission fluxes at both the soil–air and water–air interfaces in wetlands are calculated using Formula (1); positive values indicate emissions, while negative values indicate absorption [15]:

(1) $$F\;=\;\frac{d_{c}}{d_{t}}\frac{M}{V_{0}}\frac{P}{P_{0}}\frac{T_{0}}{T}H$$

In the formula: *F* represents the gas flux (mg·m^−2^·h^−1^ or μg·m^−2^·h^−1^); *V*_0_, *P*_0_, and *T*_0_ represent the molar volume, standard atmospheric pressure, and absolute temperature of a gas under standard conditions, respectively; *V*_0_ is taken as 22.4 L/mol, *P*_0_ as 101.325 kPa, and *T*_0_ as 273.15 K; *dc*/*dt* is the slope of the line representing the change in gas concentration over time during sampling; *M* is the molar mass of the gas being measured; CO_2_ is 44, CH_4_ is 16, and N_2_O is 44; *P* and *T* are the actual atmospheric pressure and air temperature inside the chamber at the sampling point; *H* is the height of the static dark chamber, 0.5 m.

(2) Cumulative GHG emissions

The average GHG emission flux for the wetland during a given period is calculated as the average of the GHG emission fluxes from two consecutive sampling days. The cumulative GHG emission flux is obtained by multiplying the average emission flux by the interval between the two sampling days and summing the results across all sampling days throughout the year. The daily GHG emission flux is calculated as the average of the GHG flux observed at a specific time on a given day. The formula for the cumulative GHG emissions is [16]:

(2) $$CE\;=\;\sum_{i=1}^{n}\frac{\left(F_{i}\;+{\;F}_{i+1})\;\times \;(t_{i+1}\;-\;t_{i}\right)}{2}\;\times \;24\;\times \;\frac{1}{1000}$$

In the formula: *CE* represents the annual cumulative emissions of greenhouse gases (CH_4_, CO_2_, or N_2_O), in g·m^−2^; *F* is the emission flux of greenhouse gases, in mg·m^−2^·h^−1^; *i* denotes the *i*-th sampling day; *t_i+_*_1_ − *t_i_* represents the time interval between two sampling days, in days; and *n* is the number of monitoring sessions per year, which is 24 for 2024–2025.

(3) Global Warming Potential

GWP is a metric used to measure the extent to which different greenhouse gases contribute to global warming. This study does not account for differences in land and water areas; instead, it simply sums the GWP values at the soil-atmosphere and water-atmosphere interfaces to assess the contribution of various wetland systems to the greenhouse effect over a 100-year timescale. On a 100-year timescale, the GWP of CH_4_ per unit mass is 28 times that of CO_2_, and the GWP of N_2_O per unit mass is 265 times that of CO_2_. The formula for calculating the GWP over a 100-year timescale is as follows [17]:

(3) $${GWP}_{s}\;={\;CO}_{2}\;\times \;1\;+{\;CH}_{4}\;\times \;28\;+\;N_{2}O\;\times \;265$$

In the formula: *GWPs* represent global warming potentials, g·m^−2^; *CO*_2_ represents cumulative emissions during the observation period, g·m^−2^; *CH*_4_ represents cumulative emissions during the observation period, g·m^−2^; *N*_2_*O* represents cumulative emissions during the observation period, g·m^−2^; 28 and 265 are conversion factors, representing the GWP multiples of CH_4_ and N_2_O relative to CO_2_ over a 100-year time scale.

### 2.5. Data Processing and Analysis

Data processing was performed using WPS Office. All statistical analyses were conducted using SPSS 27. Prior to parametric tests, data were first assessed for normality (Shapiro–Wilk test) and homogeneity of variances (Levene’s test). Paired t-tests were used to compare greenhouse gas emission fluxes between the soil–air and water–air interfaces within the same wetland and at the same time. To compare fluxes among the three different wetlands (Haihu, Ninghu, and Beichuanhe) or across different months, one-way ANOVA was performed, followed by Tukey HSD post hoc multiple comparisons. All tests were two-tailed, with a significance level set at *p* < 0.05. Figures were plotted using Origin 2021 and ArcGIS 10.8. In the figures showing greenhouse gas fluxes (CH_4_, CO_2_, N_2_O), data were presented as the mean ± standard deviation (SD) of three replicate measurements per wetland per month. When conducting Redundancy Analysis (RDA), the physicochemical properties of soil, water, and sediments were first standardized using the minimum-maximum method (scaling them to the range of 0–1). Subsequently, RDA analysis was performed using Canoco 5.0 software to identify relationships between greenhouse gas emission fluxes and these physicochemical properties. Specifically, the average greenhouse gas emission flux across different wetland interfaces was used as the response variable, while the standardized physicochemical properties of soil, water, and sediment served as explanatory variables. In the resulting RDA biplot, the angles between different line segments (vectors) reflected the strength of positive or negative correlations among the variables.

## 3. Results and Analysis

### 3.1. Characteristics of GHG Emission Fluxes Across Different Interfaces in Wetlands of Xining City

#### 3.1.1. Daily Variations in CH_4_ Emission Fluxes from Wetlands

**Figure 3:**
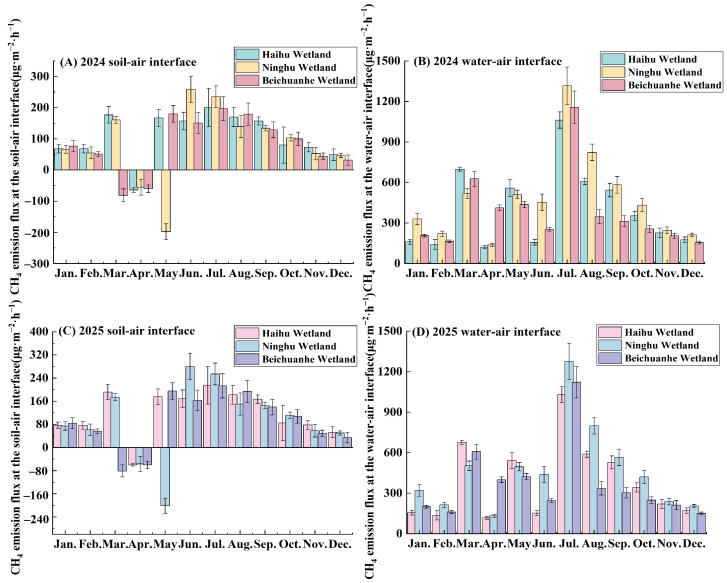
Trends in CH_4_ emission fluxes from different wetland interfaces in 2024 and 2025. Panels: (**A**) 2024 soil–air interface, (**B**) 2024 water–air interface, (**C**) 2025 soil–air interface, (**D**) 2025 water–air interface.

Figure 3 shows the characteristics of CH_4_ emission fluxes at the two interfaces in the three wetlands within the study area for 2024 and 2025. The CH_4_ emission fluxes at the water–air interface in all three wetlands were greater than those at the soil–air interface. Between 2024 and 2025, the ranges of CH_4_ emission fluxes at the water–air and soil–air interfaces were 99.5–1460.5 μg·m^−2^·h^−1^ and −231.5–330.2 μg·m^−2^·h^−1^, respectively. The soil–air interface of the Haihu Wetland exhibited the highest average CH_4_ emission flux (117.48 μg·m^−2^·h^−1^), while the water–air interface of the Ninghu Wetland exhibited the highest average CH_4_ emission flux (481.97 μg·m^−2^·h^−1^). The water–air interface in all wetlands acted as a “source” of CH_4_, while the soil–air interface served as a “sink” for CH_4_ between March and May. Specifically, all three wetlands exhibited weak CH_4_ uptake in April, with the Beichuanhe wetland showing weak CH_4_ uptake beginning in March and the Ninghu wetland in May. Seasonal variations in CH_4_ emission fluxes across different interfaces were relatively pronounced, and significant differences were observed between the two interfaces (*p* < 0.05). CH_4_ emission fluxes at both interfaces increased with rising temperatures, generally following the pattern: summer > autumn > spring > winter. Specifically, the average CH_4_ emission fluxes at the soil–air interface in spring, autumn, and winter were Haihu Wetland > Ninghu Wetland > Beichuanhe Wetland, whereas in summer, the order was Ninghu Wetland > Haihu Wetland > Beichuanhe Wetland. Regarding the average CH_4_ emission flux at the water–air interface in wetlands, the summer and fall seasons showed Ninghu Wetland > Haihu Wetland > Beichuanhe Wetland, while spring showed Beichuanhe Wetland > Haihu Wetland > Ninghu Wetland, and winter showed Ninghu Wetland > Beichuanhe Wetland > Haihu Wetland.

#### 3.1.2. Daily Variations in CO_2_ Emission Fluxes from Wetlands

**Figure 4:**
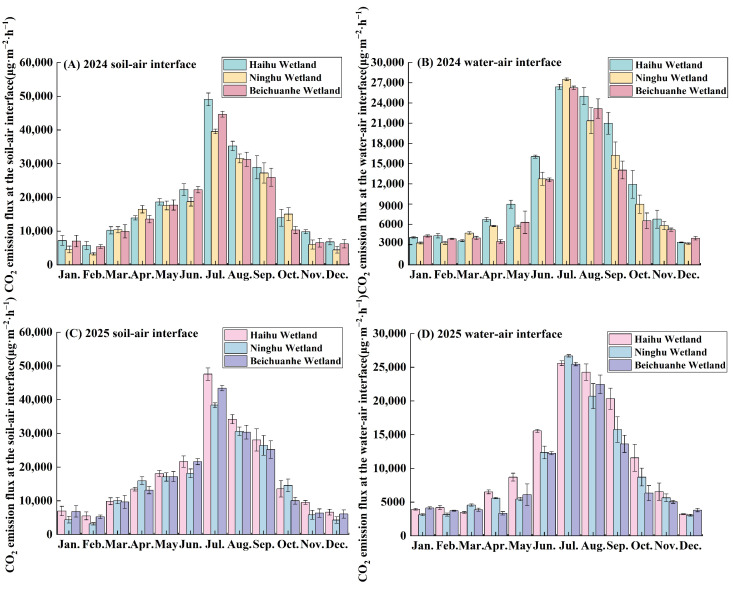
Trends in CO_2_ emission fluxes from different wetland interfaces in 2024 and 2025. Panels: (**A**) 2024 soil–air interface, (**B**) 2024 water–air interface, (**C**) 2025 soil–air interface, (**D**) 2025 water–air interface.

Figure 4 shows the characteristics of CO_2_ emission fluxes at the two interfaces in the three wetlands within the study area for 2024 and 2025. The CO_2_ emission fluxes at the soil–air interface in all three wetlands were greater than those at the water–air interface, and the differences in CO_2_ emission fluxes between the two interfaces were not significant (*p* > 0.05). Between 2024 and 2025, the ranges of CO_2_ emission fluxes at the water–air and soil–air interfaces were 2924.2–27,695.1 μg·m^−2^·h^−1^ and 2904.7–51,224.5 μg·m^−2^·h^−1^, respectively. The soil–air interface at the Ninghu Wetland exhibited the lowest average CO_2_ emission flux (15,751.06 μg·m^−2^·h^−1^), while the water–air interface at the Beichuanhe Wetland exhibited the lowest average CO_2_ emission flux (9188.05 μg·m^−2^·h^−1^). In contrast to CH_4_ emission fluxes, CO_2_ emission fluxes at all three wetlands acted as “sources,” exhibiting an overall trend of a gradual increase followed by a gradual decrease. CO_2_ emission fluxes at both interfaces peaked in July and maintained a sustained peak in August, indicating that CO_2_ emission fluxes were highest in summer and lowest in winter. Furthermore, no significant spatial differences were observed in CO_2_ emission fluxes among the three wetlands.

#### 3.1.3. Daily Variations in N_2_O Emission Fluxes from Wetlands

**Figure 5:**
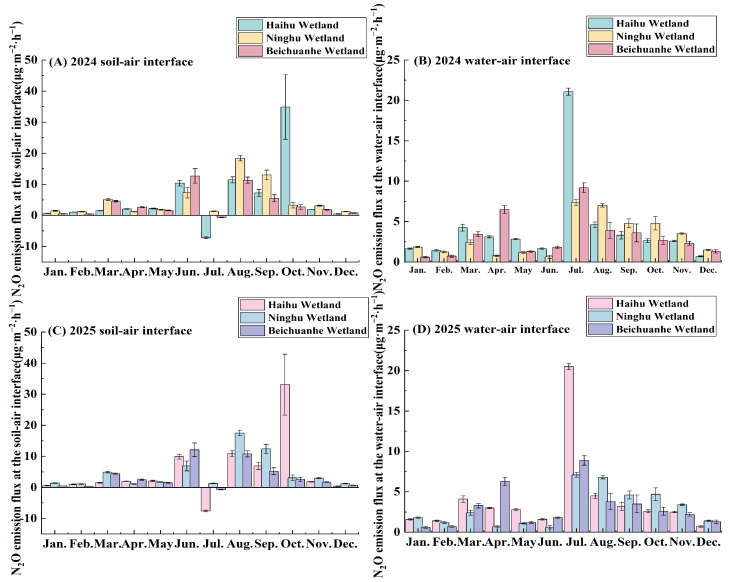
Trends in N_2_O emission fluxes from different wetland interfaces in 2024 and 2025. Panels: (**A**) 2024 soil–air interface, (**B**) 2024 water–air interface, (**C**) 2025 soil–air interface, (**D**) 2025 water–air interface.

Figure 5 shows the characteristics of N_2_O emission fluxes at the two interfaces of the three wetlands in the study area for 2024 and 2025. N_2_O emission fluxes at the soil–air interface of the wetlands exhibited an irregular trend, generally acting as either a “source” or a “sink” of N_2_O, while the water–air interface of the wetlands remained a “source” of N_2_O. Specifically, the soil–air interface of the Haihu Wetland and Beichuanhe Wetland exhibited N_2_O uptake only in July. N_2_O emission fluxes at the soil–air interface were slightly lower than those at the water–air interface, and the difference in N_2_O emission fluxes between the two interfaces was significant (*p* < 0.05). N_2_O emission fluxes at the soil–air interface reached a peak of uptake in July and a peak of emission in June and August. In contrast, the water–air interface reached a peak of emission in July and maintained a small peak of emission in August. From a seasonal perspective, N_2_O emission fluxes at both interfaces in the wetlands followed the order: summer > autumn > spring > winter. Between 2024 and 2025, the ranges of N_2_O emission fluxes at the water–air and soil–air interfaces were 0.42–21.58 μg·m^−2^·h^−1^ and −7.58–46.21 μg·m^−2^·h^−1^, respectively. The Beichuanhe wetland exhibited the lowest average N_2_O emission flux at the soil–air interface (3.47 μg·m^−2^·h^−1^), while the Haihu wetland had the highest average N_2_O emission flux at the water–air interface (4.16 μg·m^−2^·h^−1^), indicating significant spatial heterogeneity in wetland N_2_O emission fluxes.

### 3.2. Analysis of Factors Affecting GHG Emission Fluxes Across Different Interfaces in Wetlands of Xining City

The above analysis revealed that greenhouse gas emission fluxes at the three wetlands in Xining exhibited significant variations over time, with relatively minor fluctuations in emission fluxes from month to month. Therefore, the physicochemical properties of soil, water, and sediments during the wetlands’ Normal flow period (March–June), High flow period (July–October), and Low flow period (November–February) were selected as the basis for analysis. GHG emission fluxes were averaged over these four-month intervals (i.e., March–June, July–October, and November–February, respectively) to identify influencing factors. The specific RDA procedures are described in Section 2.5.

#### 3.2.1. RDA Analysis of Emission Fluxes at the Soil–Air Interface in Wetlands and Their Relationship to Soil Physicochemical Properties

(1) Physicochemical Properties of Wetland Soils

**Figure 6:**
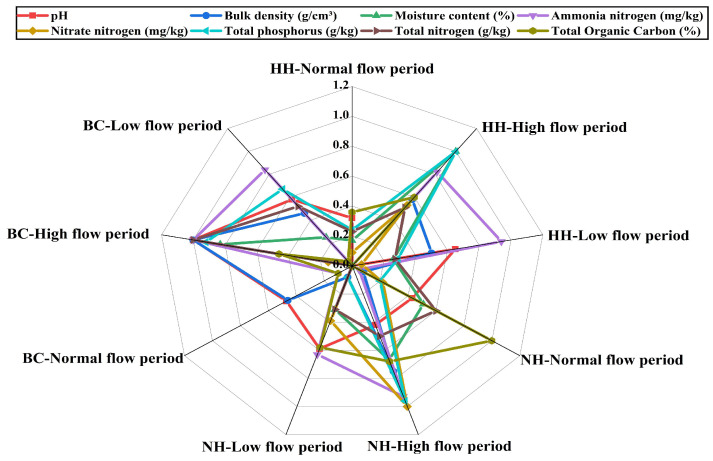
Characteristics of changes in the physicochemical properties of wetland soils at different stages.

Figure 6 shows the characteristics of soil physicochemical properties during the study period. There were minor differences in moisture content among the different wetlands. The changes in soil physicochemical properties, including NH_3_-N, NO_3_^−^-N, TP, TN, and TOC, over time were relatively pronounced. Specifically, soil NH_3_-N levels were higher during the High flow period and the Low flow period than during the Normal flow period, with the highest value observed in the Ninghu Wetland during the High flow period. Conversely, NO_3_^−^-N, TP, TN, and TOC in wetland soils peaked during the High flow period, with overall concentrations following the order: NH > HH > BC.

(2) RDA Analysis of Emission Fluxes at the Soil–Air Interface and Soil Physicochemical Properties

**Figure 7:**
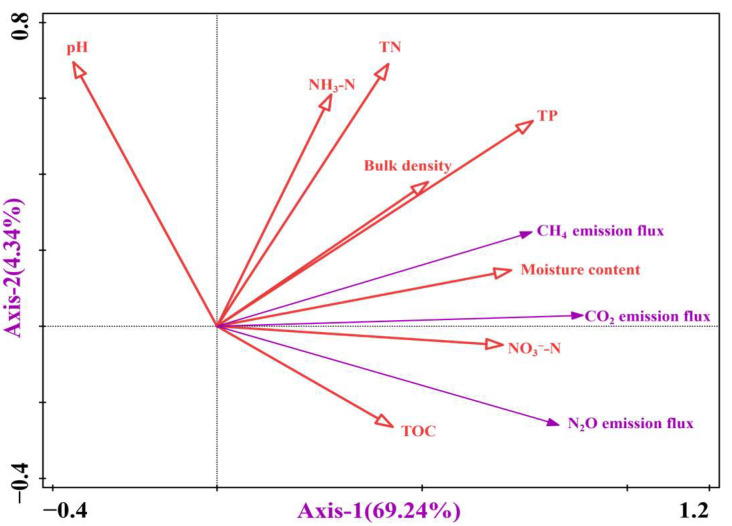
Correlation between GHG emission fluxes at the soil–atmosphere interface in wetlands and soil physicochemical properties. Note: The data in this figure represents the average of two consecutive years of monitoring in 2024 and 2025.

As shown in Figure 7, the explanatory power of environmental factors for Principal Axis 1 is 69.24%, the explanatory power of greenhouse gas emission fluxes for Principal Axis 2 was 4.34%, and the cumulative explanatory power of the two principal axes was 73.57%. The variable with the highest explanatory power was TP (*p* = 0.002, contribution rate 56%), followed by NO_3_^−^-N (*p* = 0.028, contribution rate 13.1%) (Table 3). Greenhouse gas emission fluxes at the soil–air interface in wetlands showed a negative correlation with pH, indicating that pH had a relatively minor influence on greenhouse gas emissions from wetlands. In contrast, greenhouse gas emission fluxes show positive correlations with soil bulk density, moisture content, TOC, NH_3_-N, NO_3_^−^-N, TN, and TP, indicating that these parameters have a significant impact on greenhouse gas emissions from wetlands. The correlation between each environmental factor and CH_4_ emission flux, from strongest to weakest, was moisture content > TP > bulk density > NO_3_^−^-N; for CO_2_ emission flux, it was NO_3_^−^-N > moisture content > TP > TOC; and for N_2_O emission flux, it was NO_3_^−^-N > TOC > moisture content > TP.

#### 3.2.2. RDA Analysis of Emission Fluxes at the Water–Air Interface in Wetlands and Their Relationship to Sediment and Water Physicochemical Properties

(3) Physicochemical Properties of Wetland Water

**Figure 8:**
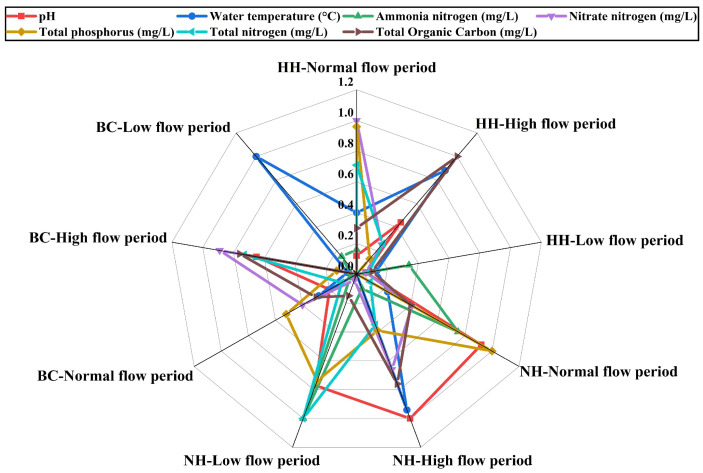
Characteristics of changes in the physicochemical properties of wetland water at different stages.

Figure 8 shows the physicochemical characteristics of water bodies in Xining during three different periods. Significant variations in water pH and temperature were observed in the wetlands of Xining across different periods. Among the NH_3_-N and NO_3_^−^-N concentrations in wetland waters during different periods, the highest NH_3_-N concentration was recorded in the NH, while the highest NO_3_^−^-N concentration was observed in the HH. Regarding the TP, TN, and TOC concentrations in wetland waters across different periods, the lowest TP and TN concentrations were both found in the BC, whereas the highest TOC concentration was recorded in the HH.

(4) RDA Analysis of Emission Fluxes at the Water–Air Interface and Water Physicochemical Properties

**Figure 9:**
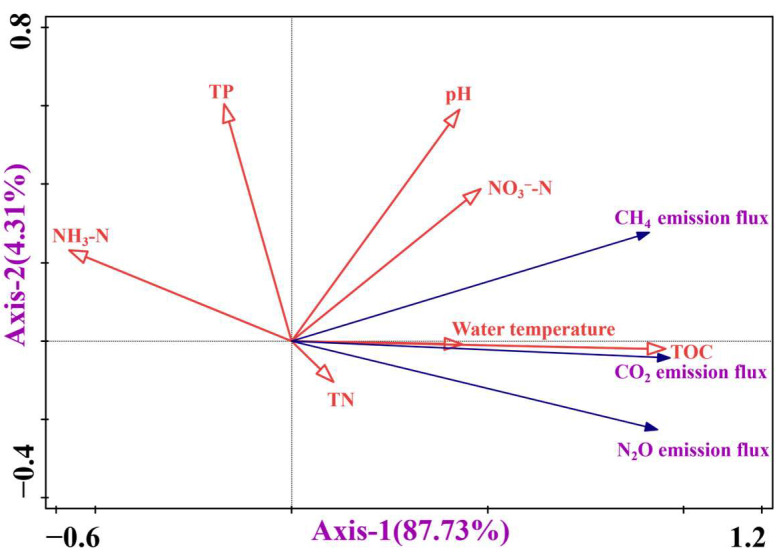
Correlation between GHG emission fluxes at the water–air interface in wetlands and the physicochemical properties of the water. Note: Same as Figure 7.

The RDA analysis of GHG emission fluxes at the water–air interface in wetlands and their relationship with aquatic environmental factors is shown in Figure 9. As shown in the figure, the explanatory power of environmental factors on Principal Axis 1 was 87.73%, and that of GHG emission fluxes on Principal Axis 2 was 4.31%; the cumulative explanatory power of both axes was 92.04%. The factor with the highest explanatory power was TOC (*p* = 0.002, contribution rate 85.8%), followed by water temperature (*p* = 0.02, contribution rate 3.3%) (Table 4). GHG emission fluxes at the wetland water–air interface were positively correlated with water temperature, TOC, pH, NO_3_^−^-N, and TN among the physicochemical properties of the water body, indicating that these indicators had a significant impact on GHG emissions. Conversely, they were negatively correlated with NH_3_-N and TP, suggesting that these two indicators had a relatively minor influence on GHG emissions. The correlation between each environmental factor and CH_4_ emission flux, from strongest to weakest, was water temperature > TOC > NO_3_^−^-N; for CO_2_ emission flux, it was TOC > water temperature > NO_3_^−^-N; and for N_2_O flux, it was TOC > water temperature > TN.

(5) Physicochemical Properties of Wetland Sediments

**Figure 10:**
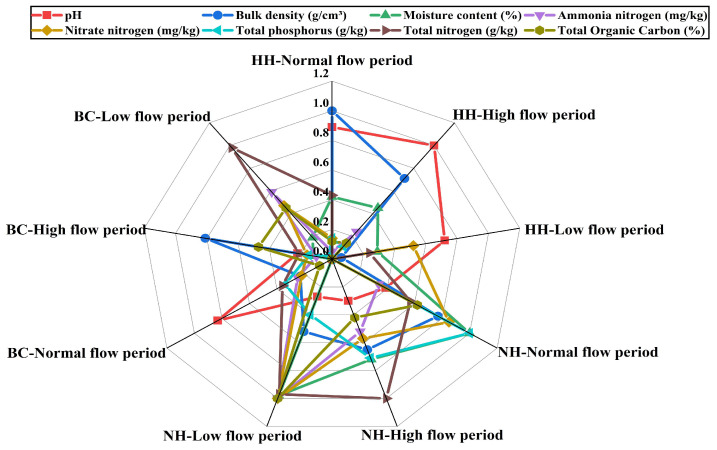
Characteristics of changes in the physicochemical properties of wetland sediments at different stages.

Figure 10 shows the physicochemical characteristics of sediments from three time periods in Xining City. For NH_3_-N and NO_3_^−^-N in sediments from different periods, NH exhibited the highest values, while for TP, TN, and TOC in sediments from different periods, the order was NH > HH > BC.

(6) RDA Analysis of Emission Fluxes at the Water–Air Interface and Sediment Physicochemical Properties

**Figure 11:**
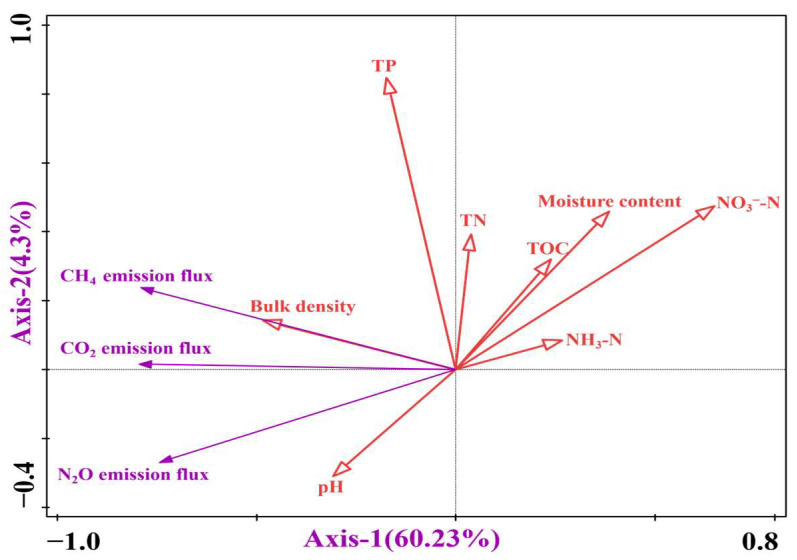
Correlation between GHG emission fluxes at the water–air interface in wetlands and the physicochemical properties of sediments. Note: Same as Figure 7.

The RDA analysis of GHG emission fluxes at the water–air interface in wetlands and sediment factors is shown in Figure 11. As shown in the figure, the explanatory power of environmental factors on Principal Axis 1 was 60.23%, and that of GHG emission fluxes on Principal Axis 2 was 4.3%; the cumulative explanatory power of both axes was 64.53%. The factor with the highest explanatory power was NO_3_^−^-N (*p* = 0.008, contribution rate 39.8%), followed by TP (*p* = 0.002, contribution rate 39.4%) (Table 5). GHG emission fluxes at the wetland water–air interface were positively correlated with pH and bulk density among the physicochemical properties of sediments, indicating that sediment pH and bulk density had a significant influence on the emissions of these three gases. The moisture content, NH_3_-N, NO_3_^−^-N, TOC, TP, and TN of sediments had a relatively minor influence on wetland GHG emissions. The correlation of various environmental factors with CH_4_ and CO_2_ emission fluxes, from strongest to weakest, was bulk density > pH > TP; for N_2_O fluxes, the order was pH > bulk density > TP.

### 3.3. Cumulative GHG Emissions and GWP in Xining’s Wetlands

GWP was used to measure the relative contribution of different greenhouse gases to global warming. By comparing the warming effect of a given gas with that of CO_2_ over a specific time period (typically 100 years), we could understand the relative impact of different gases on climate change. The average greenhouse gas flux observed in the wetlands of Xining City at a specific time on a given day (9:00 a.m. to 8:00 p.m.) could be used as an approximation of the average daily GHG flux. The cumulative emissions and wetland GWP were calculated, and the statistical results are shown in Table 6.

The interannual cumulative emissions at the soil–air interface for the three wetlands were as follows: HH 156.13 g·m^−2^, NH 137.61 g·m^−2^, and BC 14.26 g·m^−2^. The interannual cumulative emissions at the soil–air interface were: 100.4 g·m^−2^ for the HH, 87.42 g·m^−2^ for the NH, and 83.00 g·m^−2^ for the BC; the interannual cumulative emissions for the Xining Wetlands were 705.88 g·m^−2^. The GWP at the soil–air interface for Xining wetlands were 195.07 g·m^−2^, 167.77 g·m^−2^, and 169.32 g·m^−2^, respectively, while those at the water–air interface were 199.92 g·m^−2^, 203.5 g·m^−2^, and 175.77 g·m^−2^, respectively. The GWP across different interfaces in Xining’s wetlands was 1111.49 g·m^−2^. Among these, GHG emissions from the soil–air interface contributed 47.88% to the wetlands’ GWP, while GHG emissions from the water–air interface accounted for 52.12%. In Xining’s wetlands, CH_4_ contributed 32.55% to the GWP, CO_2_ contributed 62.33%, and N_2_O contributed 5.12%.

## 4. Discussion

### 4.1. A Study on GHG Emission Fluxes in Wetlands of Xining City

Globally, high-altitude plateaus (e.g., the Qinghai–Tibet Plateau, the Andean Plateau, and the Ethiopian Highlands) host numerous cities located above 2000 m [18,19]. These “plateau cities” share common environmental characteristics: low air pressure and reduced oxygen partial pressure, low mean annual temperature, large diurnal temperature ranges, and intense solar radiation [20,21,22]. Wetlands in such cities may exhibit distinct GHG emission patterns compared to lowland or temperate wetlands, yet they remain critically understudied [23,24,25]. Xining City (elevation ~2260 m) on the eastern Qinghai–Tibet Plateau is a representative example of a plateau city. The following discussion interprets our findings from Xining not only as local observations but also as a case study that can inform understanding of GHG fluxes in other plateau urban wetlands worldwide.

#### 4.1.1. CH_4_ Dynamics

Wetland sediments are typically characterized by anaerobic conditions that favor CH_4_ production and emission [26]. In the present study, the water–air interface generally acted as a CH_4_ source throughout most of the observation period, and CH_4_ fluxes exhibited clear seasonal variability, with peak emissions occurring during July. Similar seasonal patterns have been widely reported in wetland ecosystems and are commonly associated with enhanced plant growth, increased microbial activity, and stronger anaerobic conditions during the warm season [27,28,29]. In contrast, CH_4_ emissions decreased during the senescence period, likely due to declining temperatures, reduced microbial activity, and vegetation harvesting or withering [30]. Differences among wetlands further suggest that anthropogenic disturbance and nutrient inputs strongly influence CH_4_ dynamics in plateau urban wetlands. Wetlands located in highly urbanized areas or receiving wastewater inputs exhibited relatively higher CH_4_ emissions, likely because elevated nutrient availability and organic matter accumulation enhanced methanogenic activity. These findings indicate that, in addition to climatic constraints associated with high-altitude environments, human disturbance and wetland management may play important roles in regulating CH_4_ emissions in urban wetlands on the Qinghai–Tibet Plateau and potentially in other plateau cities worldwide.

#### 4.1.2. CO_2_ Dynamics

CO_2_ emissions exhibited pronounced temporal variability across the studied wetlands, with higher fluxes generally occurring during the warm season. Similar seasonal patterns have been widely reported in freshwater and riparian wetlands, where increasing temperature enhances plant root respiration and microbial decomposition of organic matter [31,32]. However, compared with many lowland wetlands, the relatively low temperatures and strong climatic fluctuations characteristic of high-altitude environments may constrain microbial activity and partially suppress CO_2_ production in plateau wetlands. In the present study, CO_2_ fluxes at the soil–air interface were generally higher than those at the water–air interface, suggesting that soil respiration and root-associated microbial processes were important sources of CO_2_ emissions [33,34]. Spatial differences among wetlands further indicate that anthropogenic disturbance, vegetation structure, and nutrient conditions can substantially influence CO_2_ dynamics in urban wetland ecosystems. Wetlands exposed to stronger human disturbance or higher nutrient inputs tended to exhibit higher CO_2_ emissions, highlighting the combined influence of environmental conditions and wetland management on greenhouse gas exchange in plateau urban wetlands.

#### 4.1.3. N_2_O Dynamics

N_2_O emissions exhibited substantial spatial and temporal variability among the studied wetlands, reflecting the strong sensitivity of N_2_O production to hydrological conditions and nitrogen cycling processes [35,36]. Wetlands influenced by wastewater inputs or elevated nutrient concentrations showed greater fluctuations in N_2_O fluxes, suggesting that anthropogenic nitrogen loading may substantially alter nitrification and denitrification dynamics in plateau urban wetlands. In addition, lower N_2_O fluxes were generally observed during the peak growing season, likely because active plant growth enhanced nitrogen uptake and stimulated the reduction in N_2_O to N_2_ through denitrification processes. Seasonal declines in temperature may further suppress microbial activity and organic matter decomposition, thereby limiting N_2_O production during colder periods [37]. Water depth also appeared to influence N_2_O dynamics, with deeper water conditions generally associated with lower emissions at the water–air interface. These findings indicate that hydrology, nutrient availability, and temperature jointly regulate N_2_O emissions in high-altitude urban wetlands.

### 4.2. A Study on Factors Affecting GHG Emissions in Wetlands of Xining City

#### 4.2.1. Impact of Wetland Soils

Wetland soil reclamation and human disturbance can substantially alter soil carbon and nitrogen distributions, microbial community structure, and biogeochemical cycling processes, thereby influencing GHG emissions [38,39]. In the present study, soil bulk density, moisture content, and nutrient availability were significantly associated with CH_4_ emissions, likely because these factors regulate soil structure, oxygen diffusion, and organic matter decomposition [40,41]. Positive relationships between TN, TP, and CH_4_ fluxes further suggest that nutrient enrichment may stimulate methanogenic activity in plateau urban wetlands. The environmental controls on CO_2_ emissions observed in this study differ somewhat from those reported for some lowland and karst wetlands [42,43]. Such differences may reflect the unique climatic conditions of high-altitude regions, where lower temperatures and stronger temperature fluctuations constrain microbial decomposition and soil respiration processes. Similar temperature-related constraints may also occur in other cold, high-altitude urban wetlands worldwide, including plateau cities in the Andes such as La Paz and Quito [20,23,24]. N_2_O emissions were also closely associated with nitrogen availability and soil environmental conditions. Previous studies across estuarine, freshwater, and alpine wetlands have similarly demonstrated that nitrogen substrates, organic carbon availability, and hydrological conditions jointly regulate nitrification and denitrification processes [44,45,46]. Our results further suggest that these controls remain important in plateau urban wetlands, where low temperature and fluctuating hydrological conditions may additionally modify microbial nitrogen cycling and greenhouse gas production.

#### 4.2.2. Impact of Wetland Water

The water bodies of the studied wetlands exhibited relatively high nitrogen and phosphorus concentrations, indicating potential eutrophication risks. Elevated nutrient concentrations may enhance algal growth, alter dissolved oxygen dynamics, and influence greenhouse gas production and exchange within wetland ecosystems [47,48,49]. Although water quality parameters remained generally stable and weakly alkaline, clear spatial and temporal variability was observed among wetlands, suggesting that hydrological fluctuations and external nutrient inputs strongly influence water physicochemical properties in plateau urban wetlands. Compared with more hydrologically stable wetlands, artificial and semi-artificial wetlands showed greater fluctuations in water level, temperature, and nutrient concentrations. Such variability may be associated with seasonal runoff, urban inflow, and differences in wetland management, which together influence nutrient cycling and biogeochemical processes. These findings suggest that anthropogenic disturbance and hydrological instability may substantially modify water environmental conditions and greenhouse gas dynamics in high-altitude urban wetlands.

#### 4.2.3. Impact of Wetland Sediment

Sediment properties also showed important spatial variability among wetlands. Sediment pH was generally weakly alkaline, consistent with previous observations from plateau lake and wetland systems [50,51]. Variations in TN, TP, NH_3_-N, and NO_3_^−^-N further indicate differences in nutrient cycling intensity and sediment biogeochemical processes among wetlands. In wetlands experiencing stronger hydrological fluctuations, alternating wetting and drying conditions may promote redox changes, sediment nutrient release, and pore water instability, thereby influencing greenhouse gas production and transport [52,53]. In contrast, wetlands with relatively stable hydrological conditions may support more continuous sedimentary environments and more stable microbial regulation of carbon and nitrogen cycling.

GHG emissions at the wetland water–air interface were strongly influenced by interactions among water chemistry, sediment properties, and hydrological conditions [54,55,56]. In the present study, CH_4_ fluxes were positively associated with nutrient concentrations in both sediments and overlying water, suggesting that nutrient enrichment and organic matter accumulation may stimulate methanogenic activity. Variations in pH and water nutrient status further indicate that multiple water environmental factors jointly regulate CH_4_ exchange at the water–air interface. CO_2_ fluxes were positively correlated with sediment bulk density and water pH, consistent with previous studies showing that organic carbon availability, dissolved oxygen conditions, and water physicochemical properties substantially influence CO_2_ dynamics in aquatic ecosystems [57,58]. These findings suggest that sediment-water interactions play an important role in regulating carbon cycling and greenhouse gas exchange in plateau urban wetlands. N_2_O emissions also showed close relationships with nutrient conditions and water physicochemical properties, although the direction and strength of these relationships differed among studies and wetland types [59,60]. Such variability likely reflects differences in hydrological regimes, nutrient loading, and redox conditions across wetland ecosystems. In the studied wetlands, strong spatiotemporal fluctuations in water environmental conditions may have altered microbial nitrogen transformation processes, thereby influencing N_2_O production and release. Overall, the concentrations of nutrients, pH, and organic matter in both water and sediments jointly regulated GHG fluxes at the wetland water–air interface [61,62].

### 4.3. A Study on Cumulative GHG Emissions and GWP in Wetlands of Xining City

#### 4.3.1. Cumulative CH_4_ Emissions

During the study period, cumulative CH_4_ emissions at the soil-atmosphere interface were lower than those reported for natural wetlands with high organic carbon content and permafrost peatland systems. This indicates that CH_4_ emissions are primarily controlled by organic substrate availability and anaerobic production conditions, while being further modulated by climate-sensitive processes such as freeze–thaw dynamics in cold-region ecosystems [63,64,65]. Across wetland interfaces, the studied systems generally acted as a net CH_4_ source, differing from sink behavior reported in some river floodplain wetlands. This difference is likely associated with variations in hydrological stability, vegetation composition, and anthropogenic disturbance, which collectively influence redox conditions and carbon substrate inputs. CH_4_ emissions were consistently higher at the water–air interface than at the soil–air interface, in agreement with patterns observed in lacustrine and coastal wetlands, where methanogenesis and bubble-mediated transport in water columns enhance emission fluxes [15,56].

#### 4.3.2. Cumulative CO_2_ Emissions

Cumulative CO_2_ emissions from both interfaces were comparable to those reported in inland lake wetland systems, but lower than those observed in highly dynamic riverine wetlands. This suggests that CO_2_ fluxes are strongly regulated by hydrological connectivity, temperature conditions, and dissolved oxygen availability. Overall, CO_2_ fluxes exceeded uptake, indicating that the wetlands functioned as net CO_2_ sources. This pattern is widely reported in managed and human-influenced wetland systems, where nutrient enrichment and hydrological alteration enhance microbial respiration and organic matter decomposition [66,67,68].

#### 4.3.3. Cumulative N_2_O Emissions

Cumulative N_2_O emissions remained relatively low and were consistent with values reported for other managed wetland ecosystems. N_2_O production is mainly governed by coupled nitrification and denitrification processes, which are highly sensitive to oxygen availability, nitrogen loading, and sediment organic matter conditions. Under fluctuating redox environments, these microbial pathways regulate N_2_O production and consumption [69,70].

#### 4.3.4. GWP of the Wetland

Regarding GWP, CO_2_ contributed the largest share, followed by CH_4_, while N_2_O contributed the least, consistent with global observations across freshwater, brackish, and coastal wetland ecosystems [71,72]. In addition, GWP at the water–air interface was higher than at the soil–air interface, indicating that aquatic pathways represent the dominant emission hotspot. Compared with other cold-region wetlands globally, the overall GWP observed in this study was relatively moderate, likely due to limited extreme thaw-driven carbon release and relatively stable hydrological conditions [16,73]. Differences among wetland types suggest that systems with higher nutrient inputs and stronger human influence tend to exhibit elevated GHG emissions, whereas wetlands with more stable waterlogging conditions tend to have lower emission intensities. Therefore, hydrological regime, nutrient availability, and anthropogenic disturbance emerge as the primary cross-scale drivers of GHG variability in urban wetland systems.

Taken together, the relative contribution of CO_2_, CH_4_, and N_2_O to GWP in Xining’s wetlands (CO_2_ > CH_4_ > N_2_O) may reflect a broader pattern in cold, high-altitude urban wetlands, although absolute fluxes remain strongly dependent on local climate, hydrological conditions, wetland management, and anthropogenic disturbance. Comparative studies across plateau cities on different continents are needed to further evaluate the generality of this pattern [74,75,76,77].

### 4.4. Limitations of the Study

This study focuses on data from the 2024–2025 period. Although it covers three key phenological phases-the regreening phase, the growing season, and the senescence phase-GHG emissions from wetlands exhibit interannual variability. Long-term, continuous, multi-site observations will help provide a more comprehensive understanding of emission patterns and their response to climate change.

Furthermore, although this study focuses on a single plateau city (Xining) and quantifies its wetland greenhouse gas emission fluxes and influencing factors, the discussion in this paper primarily centers on comparisons with wetlands in other Chinese cities and has not yet been extended to a global scale. Future research will conduct cross-regional comparative analyses with other plateau cities, including those in South America, East Africa, Central Asia, and other regions [78,79].

This study focused on the effects of conventional environmental factors on emission fluxes; however, direct monitoring of microscopic mechanisms such as microbial community structure and enzyme activity remains insufficient. Future research could incorporate techniques such as metagenomics to provide deeper insights into microbial-mediated carbon and nitrogen cycling processes.

## 5. Conclusions

This study analyzed the characteristics of GHG (CH_4_, CO_2_, and N_2_O) emission fluxes in Xining’s wetlands based on measurements taken between 2024 and 2025. It examined the influence of environmental factors (including air temperature used in flux calculations) on GHG emission fluxes in these wetlands and estimated the cumulative GHG emissions and GWP. The findings were summarized as follows:

(1) During the 2024–2025 observation period, GHG emissions from different interfaces in Xining’s wetlands generally acted as a “source.” There were significant differences (*p* < 0.05) in GHG emission fluxes across different interfaces, exhibiting an emission pattern of summer > autumn > spring > winter. GHG emission fluxes at the wetland water–air interface were greater than those at the soil–air interface. Specifically, CH_4_ fluxes at the water–air interface were consistently higher than those at the soil–air interface, with negative fluxes (sink) occurring at the soil–air interface only in spring. CO_2_ flux was higher at the soil–air interface than at the water–air interface, and all wetlands were net CO_2_ sources. N_2_O flux exhibited high spatiotemporal variability, with the water–air interface acting as a net source throughout the study period.
(2) RDA analysis of wetland environmental factors measured during the 2024–2025 observation period revealed that the primary influencing factors at the soil–air interface were TP, NO_3_^−^-N, and moisture content. At the water–air interface, the combined effects of sediments and water were examined, with the primary influencing factors being sediment NO_3_^−^-N, TP, and NH_3_-N, as well as water TOC and water temperature. Greenhouse gas emissions from Xining’s wetlands are jointly influenced by the wetland’s water-heat environment and increase or decrease with changes in ambient temperature.
(3) By aggregating the average emission fluxes obtained for Xining, the interannual cumulative emissions and GWP of Xining’s wetlands were calculated. The results showed that the interannual cumulative emissions across different interfaces in Xining’s wetlands were 705.88 g·m^−2^, and the cumulative GWP across these interfaces was 1111.49 g·m^−2^. The GWP at the soil–air interface was slightly lower than that at the water–air interface, and the GWP of CO_2_ in wetland greenhouse gases was significantly higher than that of CH_4_ and N_2_O.

## Figures and Tables

**Table 1:** Main characteristics of the three wetlands.

Characteristics	Beichuanhe Wetland	Ninghu Wetland	Haihu Wetland
Average altitude (m)	~2260		
Hydrological condition	Natural river connectivity	Artificial regulation	Semi-regulated
Human disturbance	Relatively low	Relatively high	Moderate
Main surrounding land use	Riparian green space	Urban residential/commercial	Urban park
Main ecological function	Ecological conservation	Water purification	Recreation and ecological restoration
Nutrient input characteristics	Lower nutrient loading	Urban runoff influence	Intermediate nutrient status

Information summarized from field investigation and previous studies [9,10,11,12,13].

**Table 2:** Methods for determining physical and chemical parameters and greenhouse gases.

Physical and Chemical Properties	Methods
Bulk Density of Soil and Sediments	Ring-Cutter Method
Moisture content of soil and sediments	Gravimetric method
Soil, Water, and Sediment pH	pH Meter
NH_3_-N, NO_3_^−^-N, TP, and TN in soil, water, and sediments	Flow-through chemical analyzer
TOC in Soil, Water, and Sediments	TOC/L Analyzer
Wetland Greenhouse Gases: CO_2_, CH_4_, N_2_O	GC 2010pro Greenhouse Gas Analyzer (Shimadzu, Kyoto, Japan)

**Table 3:** Explanatory power, contribution rates, and significance tests for RDA analysis of GHG emission fluxes in relation to the physicochemical properties of soils in Xining wetlands.

Name	Explains %	Contribution %	Pseudo-F	*p*
TP	42.3	56.0	18.3	0.002
NO_3_^−^-N	9.9	13.1	4.9	0.028
Moisture content	5.3	7.1	2.9	0.072
NH_3_-N	6.1	8.1	3.7	0.05
Bulk density	7.5	10.0	5.5	0.016
TN	3.3	4.4	2.6	0.086
TOC	0.6	0.8	0.5	0.65
pH	0.4	0.5	0.3	0.792

**Table 4:** Explanatory power, contribution rates, and significance tests for RDA analysis of GHG emission fluxes in relation to the physicochemical properties of Water in Xining wetlands.

Name	Explains %	Contribution %	Pseudo-F	*p*
TOC	79.8	85.8	98.5	0.002
Water temperature	3.1	3.3	4.3	0.02
pH	2.3	2.5	3.5	0.028
NH_3_-N	3.1	3.3	5.7	0.012
TN	2.1	2.3	4.5	0.024
NO_3_^−^-N	1.7	1.8	4.2	0.024
TP	0.9	1.0	2.5	0.1

**Table 5:** Explanatory power, contribution rates, and significance tests for RDA analysis of GHG emission fluxes in relation to the physicochemical properties of Sediments in Xining wetlands.

Name	Explains %	Contribution %	Pseudo-F	*p*
NO_3_^−^-N	26.4	39.8	9.0	0.008
TP	26.2	39.4	13.2	0.002
NH_3_-N	6.8	10.2	3.8	0.06
Moisture content	3.8	5.7	2.3	0.13
TOC	1.5	2.3	0.9	0.346
TN	1.4	2.0	0.8	0.404
Bulk density	0.2	0.3	0.1	0.804
pH	0.2	0.3	<0.1	0.834

**Table 6:** Cumulative GHG emissions and GWP across different wetland interfaces.

GHG Emission Interface	Wetland Name	Cumulative Interannual Emissions (g·m^−2^)			Total (g·m^−2^)	Global Warming Potential GWP_s_ (g·m^−2^)			Total (g·m^−2^)
		CH_4_	CO_2_	N_2_O		CH_4_	CO_2_	N_2_O	
Soil–air interface	HH	0.955	155.12	0.05	156.125	26.695	155.12	13.25	195.065
	NH	0.725	136.84	0.04	137.605	20.325	136.84	10.6	167.765
	BC	0.745	140.485	0.03	141.26	20.885	140.485	7.95	169.32
Water–air interface	HH	3.34	97.095	0.035	100.47	93.55	97.095	9.275	199.92
	NH	4.01	83.38	0.03	87.42	112.32	83.38	7.95	203.65
	BC	3.145	79.82	0.03	82.995	87.995	79.82	7.95	175.765
Total GHG emissions in the study area		12.92	692.74	0.215	705.875	361.77	692.74	56.975	1111.485

## Data Availability

The datasets used and/or analyzed during the current study are available from the corresponding author on reasonable request.

## Abbreviations

The following abbreviations are used in this manuscript:

HH	Haihu Wetland
NH	Ninghu Wetland
BC	Beichuanhe Wetland
